# ‘Mindful eating’ for reducing emotional eating in patients with overweight or obesity in primary care settings: A randomized controlled trial

**DOI:** 10.1002/erv.2958

**Published:** 2022-11-17

**Authors:** Héctor Morillo‐Sarto, Yolanda López‐del‐Hoyo, Adrián Pérez‐Aranda, Marta Modrego‐Alarcón, Alberto Barceló‐Soler, Luis Borao, Marta Puebla‐Guedea, Marcelo Demarzo, Javier García‐Campayo, Jesús Montero‐Marin

**Affiliations:** ^1^ University of Zaragoza Zaragoza Spain; ^2^ Research Network on Chronicity, Primary Care and Health Promotion RD21/0016/0005 RICAPPS Zaragoza Spain; ^3^ Aragon Institute for Health Research IIS Aragon Zaragoza Spain; ^4^ Department of Basic Developmental and Educational Psychology Autonomous University of Barcelona Barcelona Spain; ^5^ AGORA Research Group, Teaching, Research & Innovation Unit Parc Sanitari Sant Joan de Déu St. Boi de Llobregat Spain; ^6^ Navarra Medical Research Institute (IdiSNA) Pamplona Spain; ^7^ Mente Aberta Brazilian Center for Mindfulness and Health Promotion Department of Preventive Medicine Universidade Federal de São Paulo São Paulo Brazil; ^8^ Department of Psychiatry Warneford Hospital University of Oxford Oxford UK

**Keywords:** emotional eating, mindful eating, mindfulness, obesity, overweight, RCT

## Abstract

**Objective:**

The primary aim of this study was to analyse the efficacy of a ‘mindful eating’ programme for reducing emotional eating in patients with overweight or obesity.

**Method:**

A cluster randomized controlled trial (reg. NCT03927534) was conducted with 76 participants with overweight/obesity who were assigned to ‘mindful eating’ (7 weeks) + treatment as usual (TAU), or to TAU alone. They were assessed at baseline, posttreatment and 12‐month follow‐up. The main outcome was ‘emotional eating’ (Dutch Eating Behaviour Questionnaire, DEBQ); other eating behaviours were also assessed along with psychological and physiological variables.

**Results:**

‘Mindful eating’ + TAU reduced emotional eating both at posttreatment (*B* = −0.27; *p* = 0.006; *d* = 0.35) and follow‐up (*B* = −0.53; *p* < 0.001; *d* = 0.69) compared to the control group (TAU alone). ‘External eating’ (DEBQ) was also significantly improved by the intervention at both timepoints. Significant effects at follow‐up were observed for some secondary outcomes related to bulimic behaviours, mindful eating, mindfulness, and self‐compassion. Weight and other physiological parameters were not significantly affected by ‘mindful eating’ + TAU.

**Conclusions:**

These findings support the efficacy of the ‘mindful eating’ + TAU programme for reducing emotional and external eating, along with some other secondary measures, but no significant changes in weight reduction were observed.

## Highlights

- ‘Mindful eating’ added to treatment as usual (TAU) is more effective than TAU alone for reducing the emotional eating pattern of obese and overweight patients in primary care (PC) settings.
- The programme produced improvements in secondary outcomes such as external eating, the severity of bulimic symptoms, the frequency of binge episodes, and some mindfulness and self‐compassion facets.
- The body mass index (BMI) and other physiological variables were not significantly reduced by the ‘Mindful eating’ programme; future studies should try to overcome some methodological shortcomings of the present study.

**Abbreviations**
ARR: Absolute risk reduction
BMI: Body mass index
BITE: Bulimic investigatory test Edinburgh
CBT: Cognitive‐behavioural therapy
CI: Confidence interval
DBP: Diastolic blood pressure
DEBQ: Dutch eating behaviour questionnaire
EAT‐26: Eating attitudes test‐26 item version
FFMQ: Five facets mindfulness questionnaire
GAD‐7: Generalised anxiety disorder questionnaire
GP: General practitioner
ICC: Intra‐cluster correlation coefficient
MB‐EAT: Mindfulness‐based eating awareness training
MES: Mindful eating scale
NNT: Number needed to treat
PC: Primary care
PHQ9: Patient health questionnaire
RCI: Reliable change index
RCT: Randomized controlled trial
SBP: Systolic blood pressure
SCS: Self‐compassion scale
SD: Standard deviation
TAU: Treatment as usual
WHO: World Health Organisation

## 1 INTRODUCTION

Recent public health approaches seek to deemphasise weight loss as a main health goal, trying to reduce stigma towards those who are overweight or obese (Penney & Kirk, 2015). This is because traditional interventions mainly focussed on weight loss, have not reliably produced positive health outcomes (Brown, 2009). People experience considerable difficulties in losing weight and maintaining weight loss, and consequently, health is now considered more related to lifestyle behaviours and more independent of body weight. Nevertheless, overweight and obesity are still considered major public health concerns since they have been consistently associated with a higher prevalence of hypertension (Landi et al., 2018), depression and anxiety (Wang et al., 2019), diabetes and cancer (Kang et al., 2018), among others. The World Health Organisation estimates that worldwide obesity has nearly tripled in the last 50 years, which accentuates the need of finding effective strategies to prevent and treat these conditions.

In PC settings, a multidisciplinary approach is often required to address the complexities that the treatment of patients with overweight and obesity may involve. These interventions often include reduced calorie diet, increased physical activity, and behavioural strategies to facilitate adherence to diet and activity goals (Tronieri et al., 2019). In addition, some treatments are focussed at achieving changes in the unhealthy eating patterns of the individual. Emotional eating, which refers to overeating during dysphoric mood (Karlsson et al., 2000), is a common pattern that is, in turn, associated with weight regain, binge eating disorder, depression and poor emotion regulation skills, with studies suggesting that the treatment of overweight and obesity with this eating pattern should not focus on calorie‐restricted diets but on emotion regulation skills (Stojek et al., 2017; van Strien, 2018).

Cognitive‐behavioural therapy (CBT) has been proved effective for changing eating patterns such as emotional eating in patients with overweight and obesity (Jacob et al., 2018). Cognitive‐behavioural therapy includes different therapeutic targets, mainly related to the relation that the individual establishes with food, rather than focussing at applying restrictive diets, which have proved to be ineffective in the long‐term (Warren et al., 2017). In the last decades, new approaches to the traditional CBT have been proposed under the term of ‘third wave’ psychotherapies, which are based on the practice of mindfulness, acceptance, compassion, and spirituality, and which have proved to be effective for treating different pathologies (Kahl et al., 2012). Mindfulness‐based programs (i.e., those addressed at developing a present‐focussed, non‐judgemental awareness) teach emotion regulation skills that can promote healthier eating behaviours since they affect behaviours and mood (Mantzios & Wilson, 2015), but do not clearly lead to weight loss (Olson & Emery, 2015).

The meta‐analysis conducted by Lawlor et al. (2020) indicated that mindfulness‐ and acceptance‐based interventions are effective for changing eating behaviours, along with producing benefits in mental health outcomes such as anxiety or depression, and enhancing psychological facets such as mindfulness and self‐compassion, defined as the ability to be considerate and kind towards oneself, specifically when experiencing suffering (Neff, 2003). Moreover, some of the studies included in the meta‐analysis reported significant changes in physiological parameters such as reductions in blood pressure, fasting glucose, and triglycerides, which represents an objective outcome that underlines the therapeutic potential of ‘third wave’ psychotherapies, although most of these findings need replication (Lawlor et al., 2020; Olson & Emery, 2015).

Some programmes are specially focussed on applying the principles of mindfulness and related concepts, such as self‐compassion, on eating behaviours with the aim of promoting ‘mindful eating’, which is defined as the enjoyment of food utilising all the senses without judgement (García‐Campayo, 2017). Such eating pattern would promote the conscious choice of food, developing awareness of the differences between physical hunger and ‘emotional’ hunger, noticing the satiety signs, and eating healthily as a response to all those signals, which could produce healthier lifestyle behaviours (Kristeller & Wolever, 2011; Warren et al., 2017). Some ‘mindful eating’ programs have been studied with positive outcomes: significant reductions in binges frequency, anxiety, and depression (Kristeller & Hallett, 1999), improvements in food‐related self‐efficacy and cognitive control (Miller et al., 2012), and improvements in reward‐driven eating (Mason et al., 2016), among others. However, a recent systematic review (Grider et al., 2021) considered that there is still weak evidence on the efficacy of ‘mindful eating’ programs and that future research using high quality study designs is needed.

The main aim of this study was to evaluate the efficacy of a ‘mindful eating’ programme, added to TAU, to reduce emotional eating patterns in adult patients, aged 45–75, with overweight or obesity in Spanish PC settings, in order to promote a healthy change in the relation with food decreasing eating disorder behaviours. This age group was chosen because they constitute the most common users of the Spanish PC system, and this age range presents a high prevalence of overweight and obesity (Spanish Health Ministry, 2020). The secondary aims were to assess the possible differences between groups in eating behaviours such as external, restrained, binge, and mindful eating as well as eating disorder risk, anxiety, depression, mindfulness facets, and self‐compassion. Finally, we explored the potential differences in anthropometric, vital sign and blood test measures to determine the scope of the intervention on these physiological parameters. The main hypothesis was that ‘mindful eating’ + TAU would be more effective than TAU alone in reducing emotional eating, both posttreatment and in the 12‐month follow‐up; and following Lawlor et al. (2020), the secondary hypotheses were that, compared with TAU alone, ‘mindful eating’ + TAU could produce improvements in the eating behaviours and physiological parameters.

## 2 METHODS

### 2.1 Design

The research design was a multicentre, two‐armed, parallel, cluster randomized controlled trial with PC centres as clusters, with equal allocation rate between groups and equal cluster size. The two arms were ‘mindful eating’ + TAU and TAU alone. Four PC centres in the city of Zaragoza, Spain, were randomly allocated to one of the two study arms. The participants were assessed in three occasions: baseline, posttreatment, and 12‐month follow‐up.

### 2.2 Participants

Participants were recruited from four PC centres in the city of Zaragoza, Spain: ‘La Jota’, ‘Las Fuentes’, ‘Almozara’ and ‘Parque Goya’. The general practitioners (GPs) of those centres had received indications for offering the possibility to participate in the study to those patients who met the following inclusion criteria: (1) aged 45–75; (2) a BMI higher than 25; and (3) fluent in Spanish. If the patient met these criteria and was interested in participating, an informed consent form was offered for them to sign along with detailed information of the study procedures. Then, a researcher who is not part of the study team assessed if they presented at least two of the following three risk factors: sedentary lifestyle, poor‐quality diet, and at least two binge episodes in a week according to the Bulimic Investigatory Test Edinburgh (BITE; Henderson & Freeman, 1987). This assessment also considered the following exclusion criteria: (1) presenting any diagnosis of an illness that could affect the central nervous system (e.g., brain affectation, dementia); (2) being diagnosed with a serious psychiatric condition (e.g., schizophrenia, acute‐phase depression, drug abuse) except for anxiety and personality disorders, since these conditions are commonly related to eating disorders; (3) presenting delusional ideas or hallucinations; (4) having risk of suicide; and (5) being part of any other medical or psychological treatment focussed on changing the relation with food or on weight reduction, apart from the TAU delivered by their GP. Presenting purging behaviours did not constitute an exclusion criterion in the present study.

The sample size calculation was based on the comparison between the study groups on the primary outcome. Considering what previous studies had reported (Alberts et al., 2012), it was assumed a large effect size (standardized *d* = 0.80) of ‘mindful eating’ + TAU compared to ‘TAU alone’ on the primary outcome (the ‘emotional eating’ subscale of the Dutch Eating Behaviour Questionnaire, DEBQ; van Strien, 2002) posttreatment. Assuming a common standard deviation and accepting an alpha of 0.05 and a statistical power of 80%, with a 1:1 allocation rate, the sample size for each arm under individual randomisation was 25. Then, this number was multiplied by the intra‐cluster correlation coefficient (ICC) to calculate the minimum number of clusters (i.e., PC centres) required; the ICC was supposed to be 0.03 (Hemming et al., 2011). This resulted in one cluster per arm; however, it was decided to include 2 clusters per arm to determine the required cluster size. With the same assumptions indicated previously, supposing that the cluster size would be at most ‘n/1’, we estimated that 16 participants per cluster would be needed, which would result in 64 participants in total. Taking into consideration an expected attrition rate of 20% at 1‐year follow‐up (Nam & Toneatto, 2016), the total sample size required was established at 76 participants (around 38 per group).

### 2.3 Procedure and ethics

The study was conducted between January 2017 and May 2018. Following the abovementioned procedure, once a patient was enroled in the study, the baseline evaluation was conducted. When all the participants were recruited, the cluster randomisation was produced by an external researcher. The study allocation was blind for the GPs, who continued providing the TAU to the participants, as well as for the evaluators to ensure the single‐blind nature of the study. Restricted randomisation was applied to balance clusters, creating comparable arms in terms of the number of clusters but also in the average per capita income of the assigned population to each PC centre, since this variable is inversely related to the presence of overweight and obesity (Newton et al., 2017) and to the ability to benefit from mindfulness‐based interventions (Spears et al., 2017). Thus, the maximally homogeneous cluster pairs in terms of average per capita income at the level of PC centre was matched and randomly divided between the intervention and control arms.

All study information was confined in secure drawers with limited access. Participant codes and personal information were stored in a separate password‐protected file, and electronic data files were password‐protected and secured via advanced encryption standard. This study was approved by the ethical committee of Aragon, Spain (PI19/086; 27/04/2016). All procedures performed in this study were in accordance with the criteria of the 1964 Declaration of Helsinki and subsequent amendments. The data were treated anonymously and were only used for the purposes of the study. The confidentiality of participants was guaranteed and protected by the Spanish Organic Law on Protection of Personal Data and Guarantee of Digital Rights (3/2018 of December 7), and all relevant EU legislation on privacy and data protection. This trial (ClinicalTrials.gov NCT03927534) was performed in compliance with the study protocol, where more details of the procedures can be found (Morillo‐Sarto et al., 2019).

### 2.4 Interventions

#### 2.4.1 ‘Mindful eating’

The ‘mindful eating’ programme was a 7‐week intervention delivered once per week in 2‐h group sessions (between 8 and 12 participants per group), conducted by a clinical psychologist trained in the protocol of the intervention (García‐Campayo, 2017), which is based on other programs such as the mindfulness‐based eating awareness training (MB‐EAT; Kristeller & Hallett, 1999). Each session combines theoretical content with mindfulness practices, and between‐session tasks are prescribed every week. The main aims of the programme are developing awareness about automatic processes, being conscious of the level of hunger, learning the relationship between eating patterns and emotions, differentiating physiological hunger from external‐derived signals to eat, finding a balance that allows enjoying eating while being conscious, identifying the bodily sensations related to an excessive intake, increasing knowledge about diet and nutrition, and learning how to take a compassionate approach to deal with binge episodes. Weight loss, on the other hand, is not a primary target of the programme, although it could happen in the long term indirectly. The programme contents are summarised in Table 1.

**TABLE 1:** Outline of the ‘Mindful eating’ intervention

	Content	Practices	Home practices
1. Introduction	Brief introduction to the programme; importance of the present moment; attention and motivation.	Breathing practice. Raisin practice. Mini‐meditation.	Breathing practice. Reducing the eating rhythm.
2. Mindful eating and compassion	What can we do with our body and your mind while meditating; what is emotional eating and how to distinguish it from physical eating; using compassion to achieve change.	Body scan practice. Healing self‐touch.	Breathing practice. Mini‐meditation.Body scan.
3. Integrating consciousness	Body signals; hunger and satiety.	Mindful eating practice. Integrating consciousness practice. Self‐acceptance.	Breathing practice. Becoming aware of hunger and satiety signals.
4. Satiety and appetite values	How to structure mindfulness practice (formal and informal); eating psychoeducation.	Compassionate body scan. Personal values practice. Chocolate practice.	Breathing practice. Mini‐meditation. Stopping in the middle of the meal. Body scan.
5. Conscious choice and forgiveness	Compassionate coping; knowing when to stop eating; potluck preparing.	Conscious choice. Full stomach practice. Forgiveness meditation.	Breathing practice. Conscious movements. Becoming aware of the continuous feeling while eating until satiety.
6. New balance consciousness	My Plate; potluck meal; nutrition and emotional eating triggers (chain).	Mindful walking practice. Emotional self‐regulation. Potluck practice.	Breathing practice. Emotional eating chain. Mindful walking. Becoming aware of our feelings and their adjustment.
7. Wisdom and future	Keeping up with the knowledge; facing yourself after relapses.	Breaking the chain. Wisdom meditation. Our inner critical voice.	‐

#### 2.4.2 Treatment as usual

Participants in both study arms continued receiving their TAU, which is the treatment that GPs administer to patients with overweight or obesity. General practitioners usually address their actions at reducing the BMI to a normal range (i.e., <25), using personalised prescriptions for each case depending on the patient's motivation and disposition to change (e.g., psychoeducation, diet records, goal setting, etc.). In this case, the TAU included two interviews with the GP and a nurse, the development of a nutritional plan and some talks on health and nutrition, for a total of 7 sessions, one per week. A more detailed description of this approach can be found elsewhere (Morillo‐Sarto et al., 2019).

### 2.5 Measures

The sociodemographic information collected at baseline was age, sex, nationality, marital status, employment situation, education, and the PC centre. The primary and some secondary outcomes, along with the process measures, were assessed in the three time points: at baseline, posttreatment and 1‐year follow‐up. Anxiety and depression were assessed at baseline and follow‐up, along with some physiological parameters.

#### 2.5.1 Primary outcome

The DEBQ (van Strien, 2002) is a 33‐item self‐reported questionnaire that measures eating styles that may contribute to the development of overweight or obesity. It is composed of three subscales, each measuring a different eating style: emotional, external, and restrained eating. The ‘emotional eating’ subscale was considered the primary outcome of the present study. It assesses the degree to which the participant considers their eating to be emotionally driven and consists of 13 items that are scored in a 5‐point Likert scale; the total score of the subscale is the result of the average of the scores of its items and ranges between 1 and 5, where higher scores indicate greater tendencies towards emotional eating. The Spanish version of the DEBQ was used, which has shown good psychometric properties, including high internal consistency for the ‘emotional eating’ subscale (*α* = 0.94) (Cebolla et al., 2014).

#### 2.5.2 Secondary outcomes

The ‘external’ and ‘restrained eating’ subscales of the DEBQ were considered secondary outcomes; the first one refers to the tendency towards eating more in the presence of certain external stimuli such as the sight or smell of food, and the latter describes the tendency towards eating less than desired to lose or maintain body weight (van Strien, 2002). Both subscales include 10 items, and the scoring system and interpretation is the same that was described for the primary outcome. Both subscales present good psychometric properties in the Spanish version of the DEBQ (*α* = 0.84 for ‘external’ and *α* = 0.93 for ‘restrained’).

The BITE (Henderson & Freeman, 1987) is a 33‐item questionnaire that assesses bulimic behaviours in non‐clinical samples. It includes two subscales: ‘symptoms’, composed of 30 dichotomous items, and ‘severity’, which includes 3 items that ask about how often the respondent fasts for a whole day, uses different strategies to lose weight and binges. The total score of the ‘symptoms’ subscale ranges from 0 to 30, and the ‘severity’ subscale can range from 0 to 39, with higher scores indicating higher bulimic tendencies. In addition, item number 27 was also independently used as a measure of frequency of binging in the last month, which is also scored in a 6‐point Likert scale with higher scores indicating higher frequency to binging. The Spanish version of the BITE has been reported to present validity in assessing specific symptoms of bulimia (*α* = 0.82 for ‘symptoms’ and *α* = 0.63 for ‘severity’) (Rivas‐Moya et al., 2004).

The Eating Attitudes Test‐26 item version (EAT‐26; Garner et al., 1982) is an adaptation of the original 40‐item questionnaire addressed at assessing eating disorder risk. The EAT‐26 includes three subscales: ‘dieting’, ‘bulimia and food preoccupation’, and ‘oral control’. Each item is scored in a 6‐point Likert scale. The Spanish version of the EAT‐26 has good psychometric properties (*α* = 0.76 to 0.89) (Constaín et al., 2014).

The Generalised Anxiety Disorder questionnaire (GAD‐7; Spitzer et al., 2006) is a common measure to assess anxiety severity during the last 2 weeks. It is composed of 7 items which are rated on a 4‐point Likert scale. The GAD‐7 presents strong sensitivity and specificity rates for discriminating patients suffering from generalised anxiety disorder. The Spanish version of the questionnaire has shown appropriate psychometric properties (*α* = 0.94) (García‐Campayo et al., 2010).

The Patient Health Questionnaire (PHQ9; Kroenke et al., 2001) is a 9‐item self‐reported measure that assesses depressive symptoms during the previous 2 weeks. Each item is scored in a 4‐point Likert scale, and the total score is calculated by summing the scores of the items. It ranges between 0 and 27 (higher scores indicate greater severity). The sensitivity and specificity rates of the PHQ9 are high, and the Spanish version has shown adequate psychometric properties (*α* = 0.86) (Diez‐Quevedo et al., 2001).

#### 2.5.3 Process measures

The Mindful Eating Scale (MES; Hulbert‐Williams et al., 2014) is a 28‐item self‐reported measure that assesses the levels of mindful awareness towards eating. The following factors are evaluated: acceptance, awareness, non‐reactivity, acting with awareness, routine, and unstructured eating. Each item is rated on a 4‐point Likert scale. The score of each subscale can be calculated by summing the items; higher scores reflect higher tendencies to mindful eating. The psychometric properties of the MES have been reported to be adequate (*α* > 0.75 for all the subscales but ‘unstructured eating’).

The Five Facets Mindfulness Questionnaire‐24 (FFMQ‐SF; Bohlmeijer et al., 2011) is an adaptation of the original FFMQ that consists of 24 items addressed at evaluating the mindfulness facets of observing, describing, acting with awareness, non‐judging, and non‐reacting to the inner experience. Each item is scored on a 5‐point Likert scale. The score of each subscale can be calculated by summing its items, which results in a score between 5 and 25 for all the subscales except for ‘Observing’, which is composed of 4 items (range: 4–20). The Spanish version of the FFMQ‐24 has presented acceptable psychometric properties (*α* = 0.65 to 0.80) (Asensio‐Martínez et al., 2019).

The Self‐Compassion Scale (SCS; Neff, 2003) was used to evaluate individual's tendency to treat themselves in a compassionate way in times of difficulty. The SCS is a 26‐item questionnaire that assesses self‐kindness, common humanity, and mindfulness. The SCS uses a 5‐point Likert‐type scale, with higher scores indicating greater levels of each self‐compassion facet. The SCS Spanish version is a reliable instrument (*α* = 0.72 to 0.79) (Garcia‐Campayo et al., 2014).

#### 2.5.4 Physiological parameters

Both at baseline and in the follow‐up assessment, weight (kg), height, and waist circumference (cm) were measured the same day and just before the psychological assessment. Blood tests were conducted to evaluate the levels of total cholesterol (as well as high‐density lipoprotein (HDL) and low‐density lipoprotein), triglycerides, alanine aminotransferase, glucose, and glycated haemoglobin. The blood test was performed in the morning (8–9 am), and all participants had fasted for 8 h prior to the test. Vital signs such as diastolic blood pressure and systolic blood pressure were also assessed. To evaluate vital signs, we used a vascular screening system (VaSera VS‐1500).

### 2.6 Data analysis

Sociodemographic and clinical data at baseline were described using means and SDs for continuous variables, and frequencies and percentages for categorical variables. Treatment conditions were compared at baseline by visual inspection to ensure the success of randomisation.

The main analysis and primary endpoint were the comparison of the effectiveness of ‘Mindful eating’ + TAU versus TAU alone at posttreatment in the main outcome (i.e., DEBQ ‘emotional eating’), which was considered a continuous variable at the individual level. Multilevel mixed‐effect regression models, including subjects and clusters (PC centres) as random effect variables, were developed by means of a repeated measures design. An intention‐to‐treat basis was considered using the restricted maximum likelihood method, which is robust in the case of small or unbalanced sample sizes (Egbewale et al., 2014). Non‐standardized and unadjusted slopes were calculated along with the 95% confidence interval. The Group × Time interaction was calculated to determine the potential differences between the groups. The effect size was calculated using Cohen's *d* (*d* ≤ 0.2 indicates small effects; *d* = 0.5 moderate; *d* ≥ 0.8 large).

Secondary analyses included the comparison of the effects of the interventions on the main outcome at 1‐year follow‐up, following the same analytical strategy described above. In the same line, both study arms were compared in the secondary outcomes, process variables, and physical parameters. Sensitivity analyses of the main outcome with sex and the baseline levels of anxiety and depression as covariates were computed, considering the existing evidence regarding the potential influence that these variables imply in terms of emotional eating (Braden et al., 2018; Frayn et al., 2018). As more than 85% of participants attended >50% of the sessions we did not develop per‐protocol analyses. The clinical significance of the potential changes produced by the interventions was further explored by calculating the absolute risk reduction (ARR) and the number needed to treat (NNT), along with their respective 95% CIs. The two criteria used for considering improvement were: (1) changing to a less severe quartile in the main outcome compared to baseline; and (2) the reliable change index (RCI; Jacobson & Truax, 1991).

An alpha level of 0.05 was set, using a two‐tailed test. Data analyses were computed using STATA v17.0, and IBM SPSS v26.0 statistical software.

## 3 RESULTS

### 3.1 Participant flow and compliance

**FIGURE 1:**
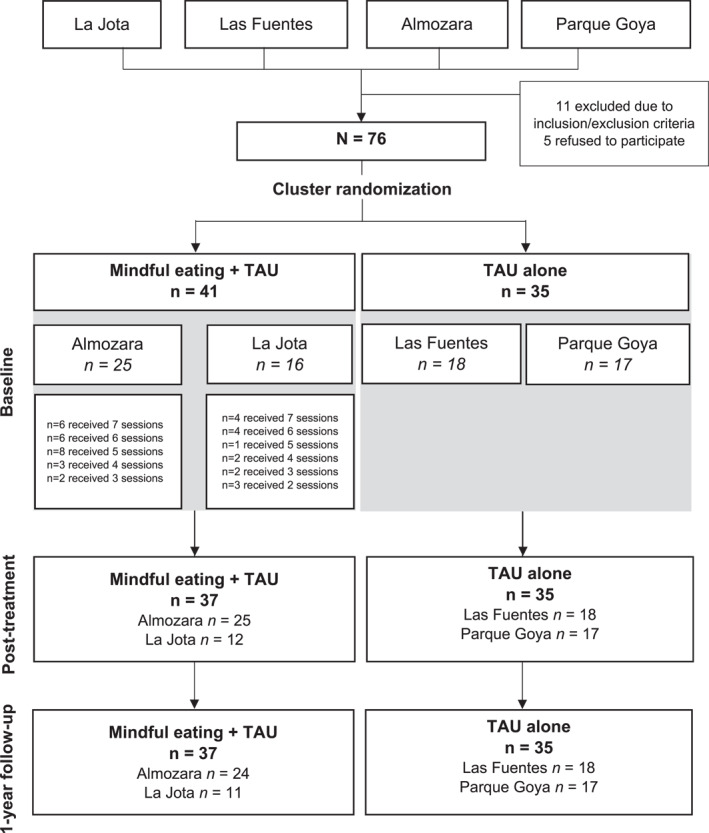
Flow chart of participants in the study

The four PC centres were randomized following the abovementioned procedure. At least 16 participants of each PC centre were enroled in the study (see Figure 1). In total, 41 participants received the intervention and 35 were allocated in the control group. In the posttreatment and follow‐up assessments, 37 (90.2%) participants in the intervention group and 100% of those in the control group provided outcome data. In the intervention group, participants attended an average of 5.20 sessions (*SD* = 1.55) out of 7; 10 patients (24.4%) attended all sessions.

### 3.2 Baseline characteristics of the study sample

Groups were similar in terms of PC centre‐related variables. The groups were also balanced in terms of sex, age, marital status, education, or employment situation. Most of the study participants were females, mostly married, and had completed primary education. The baseline characteristics of the sample, including clinical variables and physiological parameters, are summarised in Table 2. In general, participants in the ‘mindful eating’ + TAU group presented less favourable mean (SD) scores at baseline than TAU controls on DEBQ emotional eating (2.65 (0.78) versus 1.97 (0.66)).

**TABLE 2:** Baseline characteristics of primary care (PC) centres and participants by study arm

	Mindful eating + TAU (*n* = 41)	TAU alone (*n* = 35)	TOTAL (*n* = 76)
PC centre characteristics	*k = 2*	*k = 2*	*k = 4*
Socioeconomic status, mean in € (SD)	10,959 (796.20)	10,090.50 (907.22)	10,524.75 (858.54)
Patients assisted per year, mean (SD)	16,776.50 (9017.73)	16,690 (8821.86)	16,733.25 (7283.60)
Percentage of immigrant population, mean (SD)	12.44 (2.04)s	11.50 (7.78)	11.97 (4.67)
Index of dependency, mean (SD)	49.15 (3.34)	49.94 (11.50)	49.55 (6.93)

Participant sociodemographic characteristics			
Sex, *n* (%)			
Females	24 (58.5%)	24 (68.6%)	48 (63.2%)
Males	17 (41.5%)	11 (31.4%)	28 (36.8%)
Age, mean (SD	58.00 (6.65)	59.09 (8.60)	58.50 (7.58)
Marital status, *n* (%)			
Single	5 (12.2%)	2 (5.7%)	7 (9.2%)
Married	32 (78%)	26 (74.3%)	58 (76.3%)
Divorced	2 (4.9%)	4 (11.4%)	6 (7.9%)
Widowed	2 (4.9%)	2 (5.7%)	4 (5.3%)
Other	0 (0%)	1 (2.9%)	1 (1.3%)
Education level, *n* (%)			
None	0 (0%)	2 (5.7%)	2 (2.6%)
Primary	24 (58.5%)	15 (42.9%)	39 (51.3%)
Secondary	13 (31.7%)	14 (40%)	27 (35.5%)
University	4 (9.8%)	4 (11.4%)	8 (10.5%)
Employment status, *n* (%)			
Employed	14 (34.1%)	13 (37.1%)	27 (35.5%)
Self‐employed	1 (2.4%)	2 (5.7%)	3 (3.9%)
Sick leave	1 (2.4%)	1 (2.9%)	2 (2.6%)
Unemployed with subsidy	2 (4.9%)	0 (0%)	2 (2.6%)
Unemployed without subsidy	2 (4.9%)	2 (5.7%)	4 (5.3%)
Housekeeper	8 (19.5%)	7 (20%)	15 (19.7%)
Retired	9 (22%)	10 (28.6%)	19 (25%)
Permanent inability	4 (9.8%)	0 (0%)	4 (5.3%)

Participant clinical characteristics			
DEBQ, mean (SD)			
Emotional eating	2.65 (0.87)	1.97 (0.66)	2.34 (0.85)
External eating	2.89 (0.66)	2.36 (0.59)	2.64 (0.68)
Restrained eating	2.73 (0.60)	2.56 (0.80)	2.64 (0.71)
BITE, mean (SD)			
Symptoms	7.38 (5.96)	5.49 (4.33)	6.49 (5.32)
Severity	2.78 (1.79)	2.57 (1.67)	2.78 (1.73)
Frequency of binging (item 27)	2.35 (1.42)	1.97 (1.40)	2.17 (1.42)
EAT‐26, mean (SD)			
Dieting	7.93 (5.92)	10.00 (5.91)	8.89 (5.96)
Bulimia and food preoccupation	1.33 (1.80)	1.80 (2.41)	1.55 (2.11)
Oral control	2.15 (2.23)	3.00 (2.61)	2.55 (2.43)
GAD‐7, mean (SD)	3.49 (4.51)	4.00 (3.96)	3.72 (4.24)
PHQ‐9, mean (SD)	4.00 (5.34)	2.89 (2.45)	3.49 (4.27)

Participant physiological parameters			
Weight, mean in kg (SD)	89.59 (16.83)	83.89 (14.59)	86.96 (15.99)
Height, mean in cm (SD)	164.71 (10.31)	163.63 (8.21)	164.21 (9.36)
Waist circumference, mean in cm (SD)	108.68 (20.96)	100.49 (13.31)	104.85 (18.16)
BMI, mean (SD)	32.93 (4.39)	31.32 (4.86)	32.19 (4.65)
Blood test markers			
Cholesterol, mean in mg/dL (SD)	197.82 (37.43)	211.34 (34.41)	204.00 (36.46)
HDL, mean in mg/dL (SD)	55.00 (13.27)	53.91 (10.88)	54.49 (12.14)
LDL, mean in mg/dL (SD)	118.22 (31.60)	134.41 (27.43)	125.72 (30.62)
Triglycerides, mean in mg/dL (SD)	122.76 (60.06)	131.59 (66.32)	126.80 (62.69)
Alanine aminotransferase, mean in U/L (SD)	23.05 (15.58)	21.13 (10.83)	22.17 (13.56)
Glucose, mean in mg/dL (SD)	99.66 (15.17)	108.31 (40.51)	103.61 (29.66)
Glycated haemoglobin, mean in % (SD)	7.06 (0.83)	6.88 (0.89)	6.96 (0.84)
Vital signs, mean in mmHg (SD)			
DBP	82.95 (7.21)	81.83 (12.74)	82.43 (10.08)
SBP	135.39 (13.83)	140.46 (24.37)	137.72 (19.43)

Abbreviations: HDL, high‐density lipoprotein; LDL, low‐density lipoprotein.

### 3.3 Effects on the primary outcome

Sixty‐eight (89.5%) participants completed the primary outcome posttreatment. The unadjusted model showed that the intervention group had significantly decreased their scores in ‘emotional eating’ compared with the control group (*B* = −0.27; *p* = 0.006) with small effects (*d* = 0.35). At 12‐month follow‐up, the comparison reflected that the intervention group significantly reduced their scores in ‘emotional eating’ (*B* = −0.53; *p* < 0.001) with a medium effect size (*d* = 0.69). These results are detailed in Table 3.

**TABLE 3:** Unadjusted between‐group analyses for primary and secondary outcomes

	Mindful eating + TAU Mean (SD)	TAU Mean (SD)	Mindful eating + TAU versus TAU		
			*d*	*Z* (*p*)	*B* (95% CI)
DEBQ ‘Emotional eating’	*n* = 39	*n* = 33			
Baseline	2.65 (0.87)	1.97 (0.66)			
Post‐treatment †	2.40 (0.63)	2.07 (0.70)	0.35	**−2.78 (0.006)**	−0.27 (−0.46 to −0.08)
Follow‐up	2.27 (0.64)	2.19 (0.68)	0.69	**−4.17 (<0.001)**	−0.53 (−0.78 to −0.28)
DEBQ ‘External eating’	*n* = 40	*n* = 35			
Baseline	2.89 (0.66)	2.36 (0.59)			
Post‐treatment	2.77 (0.52)	2.43 (0.54)	0.31	**−2.11 (0.035)**	−0.19 (−0.036 to −0.01)
Follow‐up	2.55 (0.46)	2.51 (0.58)	0.85	**−5.68 (<0.001)**	−0.50 (−0.67 to −0.33)
DEBQ ‘Restrained eating’	*n* = 35	*n* = 34			
Baseline	2.73 (0.60)	2.56 (0.80)			
Post‐treatment	2.82 (0.62)	2.58 (0.78)	0.03	0.34 (0.735)	0.04 (−0.18–0.25)
Follow‐up	2.73 (0.50)	2.54 (0.78)	0.12	−0.26 (0.792)	−0.03 (−0.24 to 0.18)
BITE symptoms	*n* = 40	*n* = 35			
Baseline	7.38 (5.96)	5.49 (4.33)			
Post‐treatment	8.67 (4.79)	7.12 (3.87)	0.02	−0.50 (0.620)	−0.45 (−2.23–1.33)
Follow‐up	5.58 (5.40)	4.89 (3.46)	0.14	−1.30 (0.192)	−1.18 (−2.95 to 0.59)
BITE severity	*n* = 40	*n* = 35			
Baseline	2.78 (1.79)	2.57 (1.67)			
Post‐treatment	2.69 (1.53)	2.40 (1.67)	0.06	0.14 (0.888)	0.06 (−0.71–0.82)
Follow‐up	1.97 (1.80)	2.89 (2.73)	0.65	**−2.89 (0.004)**	−1.13 (−1.89 to −0.36)
BITE item 27	*n* = 40	*n* = 35			
Baseline	2.35 (1.42)	1.97 (1.40)			
Post‐treatment	2.11 (1.29)	2.00 (1.41)	0.14	−0.87 (0.385)	−0.26 (−0.84 to 0.32)
Follow‐up	1.63 (1.37)	2.20 (1.69)	0.53	**−3.26 (0.001)**	−0.96 (−1.54 to −0.38)
EAT‐26 dieting	*n* = 40	*n* = 35			
Baseline	7.93 (5.92)	10.00 (5.91)			
Post‐treatment	7.79 (6.95)	9.76 (5.93)	0.01	−0.12 (0.903)	−0.13 (−2.25–1.99)
Follow‐up	7.75 (6.09)	9.34 (5.92)	0.11	0.19 (0.852)	0.20 (−1.89–2.28)
EAT‐26 bulimia	*n* = 40	*n* = 35			
Baseline	1.33 (1.80)	1.80 (2.41)			
Post‐treatment	1.15 (1.35)	1.62 (2.22)	0.08	0.20 (0.840)	0.07 (−0.65–0.80)
Follow‐up	0.78 (1.31)	1.57 (1.96)	0.06	−0.84 (0.399)	−0.31 (−1.02–0.41)
EAT‐26 oral control	*n* = 40	*n* = 35			
Baseline	2.15 (2.23)	3.00 (2.61)			
Post‐treatment	1.97 (2.43)	2.76 (2.57)	0.08	−0.25 (0.803)	−0.10 (−0.89 to 0.69)
Follow‐up	2.00 (2.19)	2.57 (2.36)	0.02	0.33 (0.745)	0.13 (−0.64–0.90)
GAD‐7	*n* = 41	*n* = 35			
Baseline	3.49 (4.51)	4.00 (3.96)			
Follow‐up	2.66 (3.98)	2.86 (4.64)	0.07	0.26 (0.797)	0.29 (−1.93–2.52)
PHQ9	*n* = 41	*n* = 35			
Baseline	4.00 (5.34)	2.89 (2.45)			
Follow‐up	2.49 (3.39)	2.96 (4.19)	0.36	−1.48 (0.139)	−1.62 (−3.78 to 0.53)

*Note*: † Main outcome at the primary endpoint. In **bold**, statistically significant results.

After including sex and the baseline levels of anxiety (GAD‐7) and depression (PHQ9) as covariates, the model reflected a similar degree of superiority of ‘mindful eating’ + TAU versus TAU alone, both posttreatment and in the follow‐up assessment. These results, along with the rest of secondary analyses with adjusted models, are detailed in Supplementary Table 1.

### 3.4 Effects on the secondary outcomes

As shown in Table 3, ‘external eating’ (DEBQ) experienced a similar effect compared to the primary outcome; the intervention group was more effective for reducing the scores in this subscale posttreatment (*B* = −0.19, *p* = 0.035, *d* = 0.31), and in the follow‐up assessment (*B* = −0.50, *p* < 0.001, *d* = 0.85); the adjusted model (Supplementary Table 1) also reflected a similar effect. Two other secondary outcomes presented significant effects both in the unadjusted and the adjusted models: the BITE severity scale (*B* = −1.13, *p* = 0.004, *d* = 0.65), and the frequency of binging (BITE item 27) (*B* = −0.96, *p* = 0.001, *d* = 0.53) presented significant effects in favour of ‘mindful eating’ + TAU in the follow‐up assessment, while no significant differences were appreciated posttreatment. The rest of secondary outcomes did not present any significant effect.

### 3.5 Effects on the process variables and physiological parameters

Posttreatment, the only significant effect was found for the FFMQ ‘Observing’ subscale, reflecting an increase in the intervention group (*B* = 1.44, *p* = 0.022, *d* = 0.44). In the follow‐up assessment, the same effect of the intervention group was found for ‘Observing’ (*B* = 1.39, *p* = 0.027, *d* = 0.32), and for some other process variables: the MES subscales ‘Nonreactivity’ (*B* = 1.18, *p* = 0.034, *d* = 0.28) and ‘Unstructured eating’ (*B* = 0.87, *p* = 0.022, *d* = 0.30), the FFMQ ‘Nonreacting’ subscale (*B* = 1.31, *p* = 0.025, *d* = 0.40), and the SCS ‘Common humanity’ subscale (*B* = 0.37, *p* < 0.001, *d* = 0.60). These results are detailed in Supplementary Table 2 along with the rest of process variables.

The physiological parameters that experienced some significant effect in the follow‐up assessment were cholesterol (total) (*B* = 22.08, *p* = 0.040, *d* = 0.57) and HDL cholesterol (*B* = 4.92, *p* = 0.043, *d* = 0.43), both favouring TAU. No study group experienced significant reductions in the BMI. These results are presented in Supplementary Table 3.

### 3.6 Number needed to treat

Changing to a less severe quartile in the main outcome compared to the participant's baseline score was considered a criterion to define responders to the intervention. The quartiles of ‘emotional eating’ (DEBQ) at baseline were established at 1.56 (Q1), 2.50 (Q2), and 2.85 (Q3). Posttreatment, 15 individuals (44.1%) in the experimental group reduced their score to an inferior quartile. In the control group, only 2 patients (5.7%) experienced such improvement. The ARR obtained was 37.67% (95%CI = 18.87%–56.46%), with a NNT of 3 (95%CI = 1.8–5.3). In the follow‐up assessment, 17 participants (48.6%) in the experimental group experienced an improvement compared to 2 (6.1%) in the control group, which resulted in an ARR of 42.51% (95%CI = 24.06%–60.96%), with a NNT of 3 (95%CI = 1.6–4.2).

The second criterion used to calculate the NNT was the RCI, which was established at 0.72 points posttreatment. With this criterion, 5 individuals (14.7%) in the intervention group experienced a reliable improvement. In the control group, no reliable changes were appreciated. The ARR obtained in this case was 14.71% (95%CI = 2.80%–26.61%), with a NNT of 7 (95%CI = 3.8–35.7). In the follow‐up assessment, the RCI was established at 1.10 points, and in this case, 3 individuals in the ‘Mindful eating’ group (8.6%) experienced a reliable improvement. The control group, again, showed no reliable changes. A not statistically significant ARR was obtained (8.57%; 95%CI = −0.70%–17.85%), and therefore, the NNT was not calculated.

## 4 DISCUSSION

Our findings support the main hypothesis of the present study: the ‘mindful eating’ programme, added to TAU, was effective for reducing emotional eating in adults with overweight or obesity in a PC setting, compared to TAU alone. Posttreatment, the effect of the intervention was small, although significant when compared to the control group, as corroborated by the adjusted models and the significant NNT that was calculated. In the follow‐up, the effect presented a medium effect size, even after controlling variables such as sex and baseline levels of anxiety and depression that have been reported to play a role on emotional eating patterns (Braden et al., 2018; Frayn et al., 2018).

These results go in line with the ones reported in previous studies regarding the efficacy of mindfulness‐based and, particularly, ‘mindful eating’ programs on changing eating behaviours such as emotional eating. Different systematic reviews have concluded that these interventions have the potential to address problematic eating behaviours (Carrière et al., 2018; Katterman et al., 2014; Warren et al., 2017). Emotional eating, as previously explained, is a common eating pattern among people with overweight and obesity, for which it has been considered a main target of interventions (Stojek et al., 2017; van Strien, 2018). It needs to be noted, however, that not all mindfulness‐based interventions have achieved significant effects on emotional eating. Kearny et al. (2012) tested the efficacy of the Mindfulness‐Based Stress Reduction programme and found no significant effects on emotional eating. This could be possibly indicating that focussing on mindful eating rather than on the practice of mindfulness as a generic aspect could enhance the effectiveness of the intervention in this specific target.

Although the focus of the present study was the emotional eating pattern, other eating behaviours were assessed and some of them were also improved by the ‘mindful eating’ programme. That was the case of external eating, defined as the tendency towards eating more in the presence of certain external stimuli such as the sight or smell of food (Karlsson et al., 2000). The intervention significantly reduced the levels of external eating both posttreatment and in the follow‐up, and again the change in the long‐term presented a higher effect size. Previous studies have already reported significant effects of ‘mindful eating’ programs on external eating; for instance, Winkens et al. (2019) found that reductions on this eating behaviour mediated the effects of the intervention on depressive symptoms. On the other hand, in our study, bulimic behaviours were partially improved by the intervention: while bulimic symptoms did not show any significant changes, their severity and the frequency of binging was reduced at 1‐year follow‐up, in line with what previous studies have reported (Godfrey et al., 2015; Katterman et al., 2014; Kristeller et al., 2014; Kristeller & Wolever, 2011). The other eating behaviours assessed did not show any significant effects, which can be attributed to the fact that this sample presented low baseline levels of these outcomes, possibly more directly associated with other eating disorders such as anorexia and bulimia nervosa (Garner et al., 1982). The same happened for anxiety and depression, which presented low scores at baseline and did not experience any significant changes, despite being variables that have been associated with overweight, obesity, and emotional eating (Sharafi et al., 2020).

According to our results, the ‘mindful eating’ programme achieved notably higher effects in the follow‐up assessment than in the posttreatment evaluation. We hypothesise that these findings could be at least partially justified by the well‐known difficulties for implementing changes in health‐related daily habits in the short‐term (Kelly & Barker, 2016), which would explain the small effects achieved posttreatment; however, the ‘mindful eating’ programme could be slowly promoting some deeper changes in the relation that the individuals establish with food and their eating habits, resulting in more significant effects in the future. Previous studies have observed that these interventions produce effects in follow‐up assessments, although Carriere et al. (2018) observed that the average follow‐up time in the studies included in their review was 16.25 weeks, suggesting that not many studies have analysed actual long‐term effects of these interventions on eating behaviours of people with overweight or obesity.

On the other hand, short‐term effects of the ‘mindful eating’ programme were found for the mindfulness facet of ‘Observing’, which were maintained in the follow‐up assessment. ‘Observing’ is defined as the tendency to noticing or attending to internal and external experiences such as sensations, thoughts, or emotions (Baer et al., 2006). The rest of effects on process variables were only presented in the follow‐up; that was the case of ‘Nonreacting to the inner experience’, which would reflect that patients who participated in the ‘mindful eating’ programme were more capable of allowing thoughts and feelings to come and go, without getting caught up in or carried away by them (Baer et al., 2006). Both observing and nonreacting seem clearly related to the contents that were practiced during the intervention, such as being more conscious of the level of hunger, learning the difference between physiological hunger and external‐derived signals to eat, or identifying bodily sensations related to satiety (García‐Campayo, 2017). Some other process variables presented changes in the follow‐up, such as the ‘common humanity’ subscale of the SCS, suggesting that the intervention enhanced self‐compassionate tendencies in the participants, who would recognise their suffering as part of the shared human experience (Neff, 2003), which is again related to the intervention's contents (García‐Campayo, 2017). Regarding the MES, significant effects were observed in the ‘nonreactivity’ subscale –clearly related to the mindfulness facet previously described– and ‘unstructured eating’, indicating that patients reduced their tendencies to multitask while eating or snack when they felt bored (Hulbert‐Williams et al., 2014). It needs to be noted, however, that most of these effects presented small‐to‐medium effect sizes, which relativises the actual impact of the intervention on these outcomes.

Despite achieving significant changes in eating patterns, the sample who underwent the ‘mindful eating’ programme did not show a significant change in weight: while it was reduced in the 1‐year follow‐up assessment, this change was not significant compared to the control group, and the same happened with the reduction in waist circumference. It was expected that reductions in emotional eating would lead to reductions in weight (Stojek et al., 2017; van Strien, 2018); the fact that our results do not corroborate this hypothesis may be related to the relatively small sample size since this was calculated considering the effects of the intervention on the primary outcome (DEBQ ‘emotional eating’), but not on the BMI, which could possibly require a larger number of participants to present significant results. Another possibility is that the reported changes in eating patterns were partially due to the patients' expectancies and/or social desirability since they may had understood the purpose of the intervention although they were not capable of applying those concepts in their day‐to‐day. Comparing ‘mindful eating’ to another programme that addresses emotional regulation would be a possible way of overcoming this limitation in future studies.

Nevertheless, evidence regarding the efficacy of ‘mindful eating’ programs or, generally, mindfulness‐based interventions for reducing BMI is unclear; our results go in line with some previous research (Daubenmier et al., 2011; Davis, 2008; Fletcher, 2011; Forman et al., 2013; Miller et al., 2012), although one meta‐analysis has concluded that ‘third wave’ psychotherapies might have effects on weight compared to control groups (Lawlor et al., 2020). It needs to be noted that, in our study, TAU was not effective for producing a significant weight reduction, which suggests that different therapeutic approaches should be considered in PC centres for treating patients with overweight and obesity. The control group experienced significant improvements in their cholesterol levels, but the exploratory nature of the analyses conducted on biological samples in our study undermines the significance of this finding. Our study also assessed other physiological parameters for which the meta‐analysis conducted by Lawlor et al. (2020) found promising evidence, but no significant changes were observed in our case.

Some limitations of this study need to be acknowledged; first, regarding the study sample, the external validity of our findings is limited considering the relatively small sample size, which probably hindered the observation of expected changes such as reductions in the BMI, and the demographic and geographic profile of our sample. Also, patients who presented severe psychiatric disorders (e.g., schizophrenia, acute‐phase depression, drug abuse) were excluded, which could have led to a not completely representative sample of the obese and overweight patients that are treated in Spanish PC centres. Second, many of the measures that we used were self‐reported and the Spanish version of the MES was not previously validated. Regarding the interventions, the participants knew the study arm they were assigned, which may have affected their expectancies, and TAU was clearly less effective than expected, at least in terms of weight reduction. Patients in the intervention group were more exposed to treatment (i.e., ‘mindful eating’ and TAU) than those in the control group (only TAU). Participants presented low baseline levels of different outcomes, which hindered observing effects of the intervention due to potential floor effects, and participants in the ‘mindful eating’ + TAU group showed less favourable scores in emotional eating at baseline, and thus presenting a greater range of possible improvements than the control group. In addition, our study did not register the frequency of mindfulness practice during the intervention or in the follow‐up period; therefore, a hypothesis regarding the impact of home‐based mindfulness practice on the maintenance of improvements could not be tested.

In conclusion, the ‘mindful eating’ programme added to TAU seems effective for reducing the emotional eating behaviour, along with improving the external eating, the severity of bulimic behaviours, the frequency of binge episodes and some mindfulness and self‐compassion facets in adults with overweight or obesity in a PC setting. However, the changes observed in the sample's eating patterns were not matched with significant reductions in their weight. To overcome potential methodological shortcomings of the present study, future works should use larger samples and compare the ‘mindful eating’ programme with others which also address emotional eating using different strategies. If the aim is weight reduction of patients with overweight or obesity, these programs should be complemented with other strategies such as dieting and physical activity.

## CONFLICT OF INTEREST

The author declares that there is no conflict of interest that could be perceived as prejudicing the impartiality of the research reported.

## PATIENT CONSENT STATEMENT

Informed written consent was provided by all participants.

## CLINICAL TRIAL REGISTRATION

NCT03927534.

## Supporting information

Supporting Information S1

## Data Availability

The data presented in this study are available on request from the corresponding author.
