# Supplementary material for: ‘Mindful eating’ for reducing emotional eating in patients with overweight or obesity in primary care settings: A randomized controlled trial
Source: Eur Eat Disord Rev. 2022 Nov 17;31(2):303–19. doi: 10.1002/erv.2958 (PMC10100015; doi:10.1002/erv.2958)
Supplement: Supplementary file 1 — Supporting Information S1 [file ERV-31-303-s001.docx]

**Supplementary table 1.** Adjusted between-group analyses for primary and secondary outcomes.

|  | Mindful eating  + TAU  *Mean* (*SD*) | TAU  *Mean* (*SD*) | Mindful eating + TAU vs TAU | | |
| --- | --- | --- | --- | --- | --- |
|  |  |  | *d* | *Z* (*p*) | *B* (95% CI) |
| DEBQ ‘Emotional eating’ |  |  |  |  |  |
| Baseline | 2.65 (0.68) | 2.02 (0.69) |  |  |  |
| Post-treatment † | 2.43 (0.69) | 2.07 (0.69) | 0.40 | **-2.84 (.005)** | -0.27 (-0.46 to -0.09) |
| Follow-up | 2.29 (0.78) | 2.19 (0.87) | 0.65 | **-4.25 (<.001)** | -0.54 (-0.78 to -0.29) |
| DEBQ ‘External eating’ |  |  |  |  |  |
| Baseline | 2.89 (0.52) | 2.37 (0.56) |  |  |  |
| Post-treatment | 2.73 (0.53) | 2.40 (0.56) | 0.35 | **-2.13 (.034)** | -0.19 (-0.36 to -0.02) |
| Follow-up | 2.54 (0.53) | 2.52 (0.56) | 0.92 | **-5.70 (<.001)** | -0.50 (-0.67 to -0.33) |
| DEBQ ‘Restrained eating’ |  |  |  |  |  |
| Baseline | 2.76 (0.59) | 2.57 (0.66) |  |  |  |
| Post-treatment | 2.80 (0.60) | 2.57 (0.67) | 0.06 | 0.28 (.782) | 0.03 (-0.19 to 0.25) |
| Follow-up | 2.68 (0.59) | 2.52 (0.66) | 0.05 | -0.32 (.749) | -0.03 (-0.25 to 0.18) |
| BITE Symptoms ^b, c^ |  |  |  |  |  |
| Baseline | 7.34 (5.32) | 5.50 (5.93) |  |  |  |
| Post-treatment | 8.78 (5.42) | 7.36 (5.95) | 0.07 | -0.47 (.641) | -0.42 (-2.20 to 1.35) |
| Follow-up | 5.58 (5.42) | 4.90 (5.93) | 0.21 | -1.29 (.198) | -1.16 (-2.93 to 0.61) |
| BITE Severity |  |  |  |  |  |
| Baseline | 2.76 (1.94) | 2.59 (2.12) |  |  |  |
| Post-treatment | 2.65 (1.99) | 2.42 (2.12) | 0.03 | 0.17 (.869) | 0.06 (-0.70 to 0.83) |
| Follow-up | 1.95 (1.99) | 2.90 (2.12) | 0.55 | **-2.87 (.004)** | -1.12 (-1.88 to -0.36) |
| BITE item 27 ^b^ |  |  |  |  |  |
| Baseline | 2.35 (1.30) | 1.96 (1.39) |  |  |  |
| Post-treatment | 2.10 (1.33) | 1.99 (1.39) | 0.21 | -0.95 (.340) | -0.28 (-0.84 to 0.29) |
| Follow-up | 1.60 (1.36) | 2.19 (1.39) | 0.73 | **-3.36 (.001)** | -0.98 (-1.55 to -0.41) |
| EAT-26 Dieting ^a^ |  |  |  |  |  |
| Baseline | 8.03 (8.89) | 9.97 (10.57) |  |  |  |
| Post-treatment | 7.85 (9.01) | 9.94 (10.58) | 0.02 | -0.13 (.896) | -0.14 (-2.26 to 1.98) |
| Follow-up | 7.57 (8.97) | 9.31 (10.57) | 0.02 | 0.19 (.851) | 0.20 (-1.89 to 2.28) |
| EAT-26 Bulimia ^a^ |  |  |  |  |  |
| Baseline | 1.35 (2.64) | 1.76 (3.13) |  |  |  |
| Post-treatment | 1.28 (2.68) | 1.61 (3.14) | 0.03 | 0.21 (.837) | 0.08 (-0.65 to 0.80) |
| Follow-up | 0.82 (2.67) | 1.53 (3.13) | 0.10 | -0.84 (.401) | -0.31 (-1.02 to 0.41) |
| EAT-26 Oral control |  |  |  |  |  |
| Baseline | 2.20 (2.14) | 2.95 (2.33) |  |  |  |
| Post-treatment | 1.91 (2.23) | 2.76 (2.36) | 0.04 | -0.25 (.806) | -0.10 (-0.89 to 0.69) |
| Follow-up | 1.99 (2.20) | 2.53 (2.33) | 0.09 | 0.33 (.745) | 0.13 (-0.64 to 0.90) |

***Note***: † Main outcome at the primary endpoint. In **bold**, statistically significant results. ^a^ = sex resulted a significant covariate in the model. ^b^ = GAD-7 resulted a significant covariate in the model. ^c^ = PHQ9 resulted a significant covariate in the model.

**Supplementary table 2.** Unadjusted between-group analyses for process variables.

|  | Mindful eating + TAU  *Mean* (*SD*) | TAU  *Mean* (*SD*) | Mindful eating + TAU vs TAU | | |
| --- | --- | --- | --- | --- | --- |
|  |  |  | *d* | *Z* (*p*) | *B* (95% CI) |
| MES Acceptance | n = 40 | n = 35 |  |  |  |
| Baseline | 12.78 (4.17) | 14.06 (4.72) |  |  |  |
| Post-treatment | 13.03 (3.59) | 14.00 (4.67) | 0.08 | 0.63 (.531) | 0.39 (-0.84 to 1.63) |
| Follow-up | 13.64 (4.09) | 14.06 (4.98) | 0.23 | 1.62 (.105) | 1.02 (-0.22 to 2.26) |
| MES Consciousness | n = 40 | n = 35 |  |  |  |
| Baseline | 16.85 (2.72) | 17.51 (2.70) |  |  |  |
| Post-treatment | 16.84 (2.66) | 17.57 (2.81) | 0.05 | -0.21 (.836) | -0.10 (-1.03 to 0.84) |
| Follow-up | 16.92 (2.60) | 17.86 (2.77) | 0.16 | -0.80 (.423) | -0.38 (-1.32 to 0.56) |
| MES Nonreactivity | n = 40 | n = 35 |  |  |  |
| Baseline | 13.95 (3.06) | 15.29 (3.42) |  |  |  |
| Post-treatment | 14.59 (3.24) | 15.14 (3.50) | 0.31 | 1.68 (.092) | 0.93 (-0.15 to 2.02) |
| Follow-up | 14.31 (3.69) | 14.63 (3.45) | 0.28 | **2.11 (.034)** | 1.18 (0.09 to 2.27) |
| MES Routine | n = 39 | n = 35 |  |  |  |
| Baseline | 12.87 (2.47) | 12.57 (3.29) |  |  |  |
| Post-treatment | 12.14 (2.31) | 12.46 (3.36) | 0.22 | -1.33 (.184) | -0.66 (-1.63 to 0.31) |
| Follow-up | 12.17 (2.54) | 12.66 (3.47) | 0.28 | -1.64 (.101) | -0.81 (-1.79 to 0.16) |
| MES Awareness | n = 39 | n = 35 |  |  |  |
| Baseline | 11.67 (3.27) | 12.97 (2.96) |  |  |  |
| Post-treatment | 10.86 (2.99) | 12.83 (3.04) | 0.12 | -1.30 (.194) | -0.65 (-1.63 to 0.33) |
| Follow-up | 11.54 (3.17) | 12.54 (3.13) | 0.13 | 0.67 (.502) | 0.34 (-0.65 to 1.33) |
| MES Unstructured eating | n = 40 | n = 35 |  |  |  |
| Baseline | 11.60 (2.71) | 12.00 (2.97) |  |  |  |
| Post-treatment | 12.08 (2.28) | 12.00 (3.15) | 0.15 | 1.17 (.243) | 0.44 (-0.30 to 1.18) |
| Follow-up | 12.22 (2.68) | 11.74 (3.00) | 0.30 | **2.29 (.022)** | 0.87 (0.13 to 1.61) |
| FFMQ Observing | n = 38 | n = 35 |  |  |  |
| Baseline | 12.92 (3.54) | 15.29 (3.56) |  |  |  |
| Post-treatment | 14.34 (3.51) | 15.06 (3.62) | 0.44 | **2.28 (.022)** | 1.44 (0.20 to 2.67) |
| Follow-up | 14.08 (3.33) | 14.80 (3.69) | 0.32 | **2.22 (.027)** | 1.39 (0.16 to 2.62) |
| FFMQ Describing | n = 38 | n = 35 |  |  |  |
| Baseline | 16.37 (3.69) | 17.57 (3.81) |  |  |  |
| Post-treatment | 15.44 (3.64) | 17.63 (3.69) | 0.16 | -1.13 (.259) | -0.75 (-2.05 to 0.55) |
| Follow-up | 16.31 (3.51) | 17.20 (3.72) | 0.14 | 0.52 (.600) | 0.35 (-0.95 to 1.64) |
| FFMQ Awareness | n = 37 | n = 33 |  |  |  |
| Baseline | 18.38 (3.68) | 18.67 (4.24) |  |  |  |
| Post-treatment | 17.18 (3.25) | 18.71 (4.04) | 0.25 | -1.55 (.121) | -1.00 (-2.26 to 0.26) |
| Follow-up | 17.14 (3.39) | 18.37 (4.19) | 0.19 | -1.01 (.314) | -0.64 (-1.88 to 0.60) |
| FFMQ Nonjudging | n = 40 | n = 34 |  |  |  |
| Baseline | 15.40 (3.52) | 13.82 (3.83) |  |  |  |
| Posttreatment | 15.43 (3.84) | 13.65 (4.23) | 0.02 | 0.06 (.955) | 0.04 (-1.37 to 1.45) |
| Follow-up | 15.39 (3.52) | 13.60 (3.95) | 0.02 | 0.21 (.837) | 0.15 (-1.25 to 1.54) |
| FFMQ Nonreacting | n = 37 | n = 35 |  |  |  |
| Baseline | 14.46 (2.65) | 16.37 (3.64) |  |  |  |
| Posttreatment | 16.03 (2.46) | 16.89 (3.60) | 0.33 | 1.65 (.098) | 0.98 (-0.18 to 2.13) |
| Follow-up | 16.00 (2.16) | 16.56 (3.06) | 0.40 | **2.23 (.025)** | 1.31 (0.16 to 2.46) |
| SCS Self-kindness | n = 38 | n = 32 |  |  |  |
| Baseline | 2.96 (0.54) | 2.98 (0.46) |  |  |  |
| Post-treatment | 2.96 (0.52) | 3.01 (0.45) | 0.15 | -0.53 (.596) | -0.06 (-0.27 to 0.16) |
| Follow-up | 2.98 (0.65) | 3.06 (0.43) | 0.29 | -0.72 (.472) | -0.08 (-0.29 to 0.13) |
| SCS Humanity | n = 39 | n = 34 |  |  |  |
| Baseline | 3.06 (0.53) | 3.34 (0.49) |  |  |  |
| Posttreatment | 3.16 (0.60) | 3.35 (0.54) | 0.06 | 0.50 (.617) | 0.05 (-0.15 to 0.23) |
| Follow-up | 3.31 (0.72) | 3.19 (0.40) | 0.60 | **3.62 (<.001)** | 0.37 (0.17 to 0.57) |
| SCS Mindfulness | n = 39 | n = 34 |  |  |  |
| Baseline | 3.08 (0.60) | 3.29 (0.49) |  |  |  |
| Post-treatment | 3.18 (0.48) | 3.28 (0.49) | 0.12 | 0.93 (.352) | 0.09 (-0.10 to 0.29) |
| Follow-up | 3.19 (0.58) | 3.26 (0.51) | 0.30 | 1.37 (.171) | 0.14 (-0.06 to 0.33) |

***Note***: In **bold**, statistically significant results.

**Supplementary table 3.** Unadjusted between-group analyses for physical parameters.

|  | Mindful eating + TAU  *Mean* (*SD*) | TAU  *Mean* (*SD*) | Mindful eating + TAU vs TAU | | |
| --- | --- | --- | --- | --- | --- |
|  |  |  | *d* | *Z* (*p*) | *B* (95% CI) |
| Weight | n = 41 | n = 35 |  |  |  |
| Baseline | 89.59 (16.83) | 83.89 (14.59) |  |  |  |
| Follow-up | 88.71 (18.46) | 83.59 (13.24) | 0.04 | -0.44 (.661) | -0.57 (-3.14 to 1.99) |
| Waist circumference | n = 40 | n = 35 |  |  |  |
| Baseline | 108.68 (20.96) | 100.49 (13.31) |  |  |  |
| Follow-up | 105.71 (14.77) | 100.46 (10.33) | 0.01 | -0.17 (.863) | -0.30 (-3.74 to 3.13) |
| BMI | n = 41 | n = 35 |  |  |  |
| Baseline | 32.93 (4.39) | 31.32 (4.89) |  |  |  |
| Follow-up | 32.11 (4.51) | 31.02 (4.14) | 0.17 | -1.31 (.189) | -0.73 (-1.83 to 0.36) |
| Cholesterol (total) | n = 38 | n = 32 |  |  |  |
| Baseline | 197.82 (37.43) | 211.34 (34.41) |  |  |  |
| Follow-up | 199.89 (38.70) | 190.96 (35.50) | 0.57 | **2.05 (.040)** | 22.08 (0.99 to 43.18) |
| LDL | n = 37 | n = 32 |  |  |  |
| Baseline | 118.22 (31.60) | 134.41 (27.43) |  |  |  |
| Follow-up | 120.59 (33.04) | 122.57 (31.31) | 0.45 | 1.60 (.109) | 14.06 (-3.11 to 31.22) |
| HDL | n = 37 | n = 32 |  |  |  |
| Baseline | 55.00 (13.27) | 53.91 (10.88) |  |  |  |
| Follow-up | 54.19 (12.21) | 48.40 (8.61) | 0.43 | **2.02 (.043)** | 4.92 (0.15 to 9.69) |
| Triglycerides | n = 38 | n = 32 |  |  |  |
| Baseline | 122.76 (60.06) | 131.59 (66.32) |  |  |  |
| Follow-up | 120.89 (44.14) | 131.25 (56.62) | 0.08 | 0.20 (.841) | 2.49 (-21.87 to 26.85) |
| Alanine aminotransferase | n = 38 | n = 32 |  |  |  |
| Baseline | 23.05 (15.58) | 21.13 (10.83) |  |  |  |
| Follow-up | 24.45 (15.39) | 25.04 (20.44) | 0.23 | -0.71 (.479) | -2.87 (-10.80 to 5.07) |
| Glucose | n = 38 | n = 32 |  |  |  |
| Baseline | 99.66 (15.17) | 108.31 (40.51) |  |  |  |
| Follow-up | 102.21 (16.41) | 108.89 (23.80) | .021 | 0.75 (.451) | 4.42 (-7.07 to 15.91) |
| Glycated hemoglobin | n = 7 | n = 8 |  |  |  |
| Baseline | 7.06 (0.83) | 6.88 (0.89) |  |  |  |
| Follow-up | 6.18 (1.00) | 6.79 (1.00) | 0.05 | -0.25 (.800) | -0.02 (-0.18 to 0.14) |
| DBP | n = 41 | n = 35 |  |  |  |
| Baseline | 82.95 (7.21) | 81.83 (12.74) |  |  |  |
| Follow-up | 78.20 (10.10) | 82.19 (10.08) | 0.06 | -0.34 (.735) | -1.42 (-9.62 to 6.79) |
| SBP | n = 41 | n = 35 |  |  |  |
| Baseline | 135.39 (13.83) | 140.46 (24.37) |  |  |  |
| Follow-up | 135.29 (15.44) | 141.95 (18.82) | 0.38 | -1.63 (.103) | -4.89 (-10.76 to 0.99) |
